# Supplementary material for: Transfer of Stress Resilient QTLs and Panicle Traits into the Rice Variety, Reeta through Classical and Marker-Assisted Breeding Approaches
Source: Int J Mol Sci. 2023 Jun 27;24(13):10708. doi: 10.3390/ijms241310708 (PMC10341874; doi:10.3390/ijms241310708)
Supplement: Supplementary file 1 [file ijms-24-10708-s001.zip › ijms-2335847-supplementary.pdf]

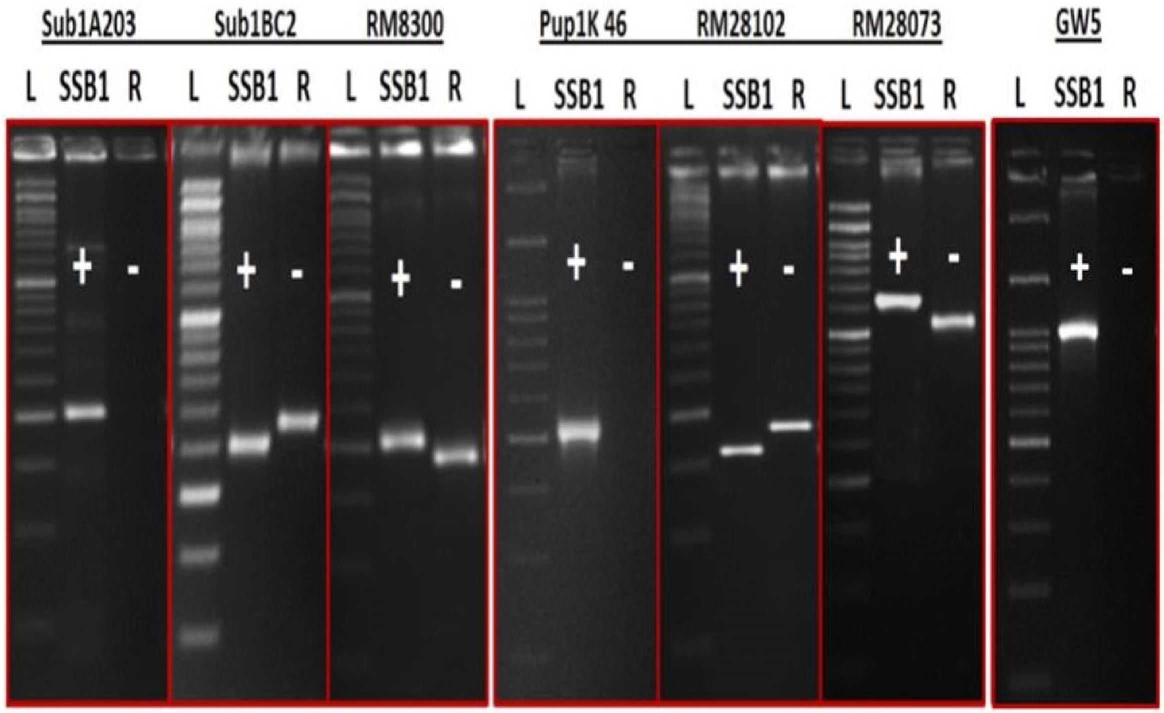

Supplementary Figure S1. PCR amplification of *Sub1*, *Pup1,* and *GW5* by deploying markers, Sub1-A203 and Sub1-BC2 for submergence tolerance; Pup1K46, RM28102 & RM28073 for low phosphorus tolerance and *GW5* for grain weight. L: Molecular weight marker (50 bp plus ladder); SSB1: Swarna-Sub1and R: Reeta.

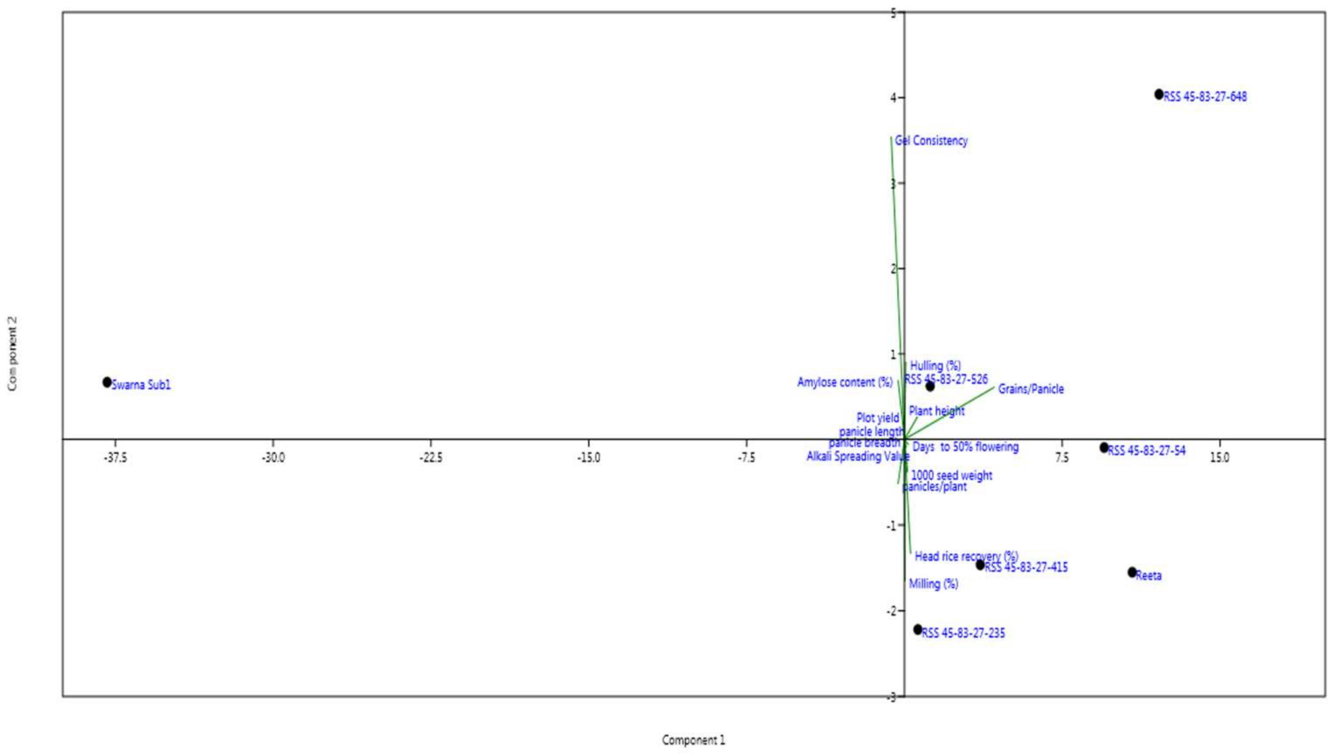

Supplementary Figure S2. Genotype-trait biplot diagram of 5 pyramided lines carrying *Sub1*, *Pup1* and *GW5* (narrow-grain) alleles along with the parents (Reeta and Swarna-Sub1) for the 14 morpho-quality traits plotted in the first two principal components.

Supplementary Table S1. Molecular markers were used for screening of submergence, low phosphorus stress tolerance, and yield component QTLs for the foreground selection.

Sl. No.	Trait name	QTL	Ch. No	Position (bp)	Primer Name	Primer sequence	Reference
1	**Submergence tolerance**	*Sub1*	9	180	RM8300	(F)5'-GCTAGTGCAGGGTTGACACA-3'<br>(R) 5'-CTCTGGCCGTTTCATGGTAT-3'	[6, 9, 17]
				200	Sub1A203	(F)5'-CTTCTTGCTCAACGACAACG-3'<br>(R) 5'-AGGCTCCAGATGTCCATGTC-3'	
				250	Sub1BC2	(F)5'-AAAACAATGGTTCCATACGAGAC-3'<br>(R) 5'-GCCTATCAATGCGTGCTCTT-3'	
2	**Low Phosphorus tolerance**	*Pup1*	12	523	Pup1K46	(F)5'-TGAGATAGCCGTCAAGATGCT-3'<br>(R) 5'-AAGGACCACCATTCCATAGC-3'	[20, 21, 25]
				168	RM28102	(F)5'-CACTAATTCTTCGGCTCCACTTTAGG-3'<br>(R) 5'-GTGGAAGCTCCGAGAAAGTGC-3'	
				656	RM28073	(F)5'-GTGTTGGTGGTGATGAAGCAAGG-3'<br>(R) 5'-GGACGAAGGATGTATGTGTCTGTACC-3'	
3	**Grain weight**	*GW5*	5	1050	GW5	(F)5'-GCGTCGTCAGAGGTAGA-3'<br>(R) 5'-GACCTAACCCATCTCATTCCA-3'	[9, 26]

Supplementary Table S2. Polymorphic SSR markers obtained between the rice varieties, Reeta and Swarna-Sub1

Chrom #	No. of markers tested	No. of polymorphic markers	Name of polymorphic markers
1	85	23	RM1003,RM10346,RM10694,RM11239,RM403,RM1349,RM212,RM220,RM297,RM5,RM6703,RM522,RM488,RM493,RM495,RM11701,RM237,RM488,RM11701,RM237,RM10793,RM24,RM312
2	68	28	RM233, RM154, RM106, RM1335, RM13439, RM211, RM263, RM341,RM6247, RM240, RM5699, RM322, RM327, RM3421, RM423, RM452,RM5897, RM492, RM6374, RM6378, RM53, RM561, RM5631, RM6318, RM6378, RM6641, RM7575, RM12808
3	69	19	RM1278, RM14723, RM175, RM293, RM251, RM520, RM3392, RM517,RM3686, RM564, RM85, RM5626, RM168, RM570, RM571, RM218, RM3329, RM2334, RM232
4	50	16	RM3735, RM5586, RM5687, RM518, RM401, RM471, RM261, RM3648,RM5424, RM3423, RM5749, RM16770, RM16686, RM1113, RM127, RM8213
5	56	8	RM18452, RM18451, RM200, RM7452, RM1089, RM592, RM440, RM574
6	57	11	RM19367, RM225, RM439, RM314, RM204, RM585, RM510, RM469, RM589, RM400, RM20377
7	66	9	RM5436, RM10, RM432, RM1209, RM3753, RM11, RM5793, RM1132, RM336
8	48	17	RM407, RM544, RM72, RM5485, RM256, RM22590, RM483, RM22585, RM22565, RM342,RM8271, RM6356, RM152, RM310, RM547, RM44, RM339
9	44	8	RM566, RM107, RM3808, RM245, RM242, RM205, RM219, RM434
10	41	9	RM311, RM179, RM271, RM6100, RM216, RM25181, RM258, RM590, RM222
11	64	5	RM202, RM536, RM209, RM4112, RM1233
12	85	15	RM5746, RM6947, RM519, RM235, RM415, RM20A, RM1246, RM17, RM1103,RM28759, RM3331, RM511, RM1261, RM28438, RM28073
Total	728	168	
